# Beneficial Effects of Sodium Nitroprusside on the Aroma, Flavors, and Anthocyanin Accumulation in Blood Orange Fruits

**DOI:** 10.3390/foods11152218

**Published:** 2022-07-26

**Authors:** Zhong-Wei Zhang, Han Liu, Hao Li, Xin-Yue Yang, Yu-Fan Fu, Qi Kang, Chang-Quan Wang, Ming Yuan, Yang-Er Chen, Shu Yuan

**Affiliations:** 1 College of Resources, Sichuan Agricultural University, Chengdu 611130, China; zzwzhang@126.com (Z.-W.Z.); lh2840806775@163.com (H.L.); lh1136820@outlook.com (H.L.); yang16970319@163.com (X.-Y.Y.); stefanlife@126.com (Y.-F.F.); kqsicauedu@163.com (Q.K.); zzwzhang@sicau.edu.cn (C.-Q.W.); 2 College of Life Science, Sichuan Agricultural University, Ya’an 625014, China; yuanmingsicau@126.com (M.Y.); anty9826@163.com (Y.-E.C.)

**Keywords:** *Citrus sinensis* (L.) Osbeck, enzyme activity, flavonoids, fruit quality, nitric oxide donor

## Abstract

The quality of Tarocco blood orange (*Citrus sinensis* (L.) Osbeck), which has been cultivated for many years, has degraded substantially. Decreased sugar content, decreased blood color, and increased sour flavor have developed as a result. To improve fruit quality, we studied the effects of bagging and sodium nitroprusside, as a nitric oxide (NO) donor, on the fruit quality of Tarocco blood orange two months before picking. The results showed that NO treatment effectively improved the content of total soluble solids and limonene in the fruit, as well as the color and hardness of the fruit, but reduced the tannin content. It also increased the contents of soluble sugar, fructose, sucrose, vitamin C, amino acids, and mineral elements. NO treatment inhibited the activities of polygalacturonase and pectin esterase, delayed the degradation of protopectin, and promoted the accumulation of anthocyanins, total flavonoids, and flavonoids synthesis. Thus, NO treatment improved the aroma, flavors, and physical properties of blood orange fruit.

## 1. Introduction

Tarocco blood orange (*Citrus sinensis* (L.) Osbeck) is rich in anthocyanins, flavonoids, and other phenolic substances that contribute to the specific color or taste of the fruit [1]. These substances are also of interest in the human diet, due to their antioxidant activities [2]. Consumers are primarily looking for the inner color of the fruit in blood oranges because the pigment is associated with health benefits [3]. However, the quality of blood oranges has degraded over long-term cultivation in some countries, resulting in a less sweet and more sour taste, as well as decreased blood color [4,5]. Compared with some high-quality sweet oranges, the flavor, quality, market price, and economic benefits of blood orange fruit are low. Therefore, improving blood orange fruit quality and increasing economic income through chemical methods are worth further research.

Nitric oxide (NO) is a highly active substance that acts as an antioxidant and biological messenger in plant systems. NO is a signal molecule with multiple functions, such as regulating flowering transition [6], promoting photosynthesis [7], and participating in fruit ripening [8,9]. The fruit ripening process is related to reactive oxygen species (ROS) production and increased lipid peroxidation. Exogenous NO prevents the elevation of H_2_O_2_ levels and lipid peroxidation, and it combines with 1-aminocyclopropane-1-carboxylate (ACC) oxidase (ACO) to form a binary complex (ACO-NO), which further combines with ACC to produce a stable ACC-ACO-NO complex. ACC-ACO-NO inhibits the increase in abscisic acid (ABA) and ethylene levels [10,11], delays fruit senescence, inhibits the activity of cell wall softening enzymes (polygalacturonase, pectin methylesterase, and pectate lyase), delays pectin degradation, and maintains fruit firmness [8].

The main components of fruit sweetness come from fructose and sucrose. It was previously reported that NO inhibited the activity of cellular glycolytic enzymes and ATP synthase through S-nitrosylation modification and reduced the activities of acetyl coenzyme A, ATP, ADP-glucose, and UDP-glucose. This ultimately led to the inhibition of polysaccharide biosynthesis, the accumulation of monosaccharides, and the increased sweetness of Arabidopsis plants [12]. NO also increased the concentration of sugars in pear and papaya [8].

Anthocyanins are important for the color development of mature fruit. It has been reported that NO could inhibit anthocyanin degradation by changing the normal structure of prophenoloxidase (PPO) active sites in fruit, inhibiting PPO activity, increasing the activities of enzymes in the anthocyanin synthesis pathway, and promoting the production of flavonoids and the accumulation of anthocyanins [13,14].

Currently, NO treatment is widely used to maintain postharvest fruit quality. NO could delay the quality reduction of postharvest mango [15], Japanese plum [16], and grape [17]. However, whether it can be further applied to fruit trees to improve fruit quality is still unknown. Nitric oxide is a byproduct of nitrate reduction [6,7]. In addition to direct NO treatments, nitrate treatments on different fruits have been also documented. For example, pre-harvest nitrate application provided adequate storage N to apple trees with no detriment to fruit quality [18]. The optimal mixed nitrogen nitrate and ammonium treatments increased fructose levels in grape berries [19].

NO treatment has been shown to improve sugar accumulation and limit anthocyanin degradation [8,12,13,14], which supports our strategy to improve the quality of blood orange fruit by NO treatments on living blood orange trees. In addition, the mechanisms by which NO improves fruit flavors were further explored in this study.

## 2. Materials and Methods

### 2.1. Blood Orange Fruits and Sodium Nitroprusside (SNP) Treatments

Tarocco blood oranges (*Citrus sinensis* (L.) Osbeck) were grown in Sichuan, China (30.01° N, 103.59° E). Blood orange fruit was treated two months before harvest (November 2020–January 2021). The experimental materials were fruit trees with the same growing conditions, and the fruit size, light, and color were similar. Fruits without visible physical damage or signs of pathogen infection were selected for treatment.

Fruits growing on the top, middle, and lower branches were selected. The fruits were divided into three groups. The first group was the control group, the second group was bagged, and the third group was treated with 5–50 μmol/L of sodium nitroprusside (SNP, a NO donor) and bagged. The paper bag contained a syringe filled with SNP solution (Figure S1). The SNP solution decomposes under light conditions to produce NO gas. The paper bags were waterproof, insect-proof, and antibacterial. The optimal concentration of 20 μM SNP was determined according to the red intensity value of the pulp analyzed by ImageJ software (NIH, Bethesda, MD, USA) [20].

### 2.2. Determination of Physical Traits of the Fruits

Fruit weights were determined with a digital balance (accuracy of 0.001 g). The fruit firmness was measured with a hardness tester (Model No. GY-3, AiPu Comp., Quzhou, China), according to the instruction manual. After removing the peel, the fruit was measured at the equator, as well as the upper and lower sides, and expressed in kg/cm^2^. Fruit size was measured by an electronic digital vernier caliper (accuracy of 0.01 mm). The fruit shape index is the ratio of longitudinal diameter to transverse diameter. The fruit samples were dried at 105 °C for 30 min and then dried to constant weight at 80 °C with a hot-air fruit-vegetable dehydrator (Model CT-C-III, Qiaoxing machinery and equipment comp., Chongqing, China). The relative water content was calculated [21]. The moisture content of the fruits decreased from 88% to about 10% after drying.

### 2.3. Determination of Flavor Compounds

The total soluble solid (TSS) content in pulp was determined at 25 °C, according to the refractive index measured with a digital refractometer (Model DP0101, Ningbo Better Technology Comp., Ningbo, China), and the result was expressed in °Brix. Titratable acidity (TA) in pulp was determined by titration with 0.1 M NaOH [22]. The absorbance of the tannin extract in the pulp was measured with a UV–Vis Spectrophotometer (DU-8600R, Drawell Instrument Comp., Chongqing, China) at a wavelength of 525 nm, and the tannin content was expressed as tannic acid equivalents [23]. The determination of limonene in the peel was carried out according to the method of Bai et al. [24]. The absorbance of the naringin extract in the pulp was measured with a UV–Vis Spectrophotometer (Drawell DU-8600R) at a wavelength of 283 nm, and the contents of naringin in the pulp were calculated by the standard curve [25].

### 2.4. Determination of Sugars and VC

The contents of soluble sugars, glucose, fructose, and sucrose in pulp were determined according to the method of Zhang et al. [26]. Vitamin C (VC) content in pulp was obtained by titration with 2,6-dichlorophenol indoxyl solution, and the results were expressed as μg of ascorbic acid per 100 g of juice [27]. The sugar–acid ratio of fruit was used to measure the flavor of fruit. It is defined as the ratio of soluble sugars to titratable acids: sugar-acid ratio = soluble sugars (%)/titratable acids (%).

### 2.5. Determination of Organic Acids and Amino Acids

Organic acids in blood orange pulp were determined by the method of Wu et al. [28]. Organic acid content was measured using an HPLC system (Model 1260, Agilent, Santa Clara, CA, USA) equipped with a Spursil C18 column (250 × 4.6 mm, 5 μm). HPLC analysis was conducted using 20 μL of the diluted supernatant. Phosphate buffer (25 mM, pH 2.5) was the mobile phase, with a flow rate of 0.8 mL/min, and the organic acids were detected at 210 nm by a diode array detector (Agilent).

Amino acids in the flesh of blood orange pulp were determined according to the method of Wistaff et al. [29]. The sample was extracted at room temperature for 1 h under the action of 0.5 mL of 0.1 M HCl and then centrifuged at 12,000 rpm for 10 min, and the supernatant was collected. Using the method published by Bao et al. [30], 10 μL of diluted supernatant was injected for UPLC analysis. The chromatographic column was a Waters BEH C18 (50 × 2.1 mm, 1.7 μm), maintained at 55 °C. The injection volume was 1 μL and the flow rate was 0.5 mL/min. Ultrapure water (phase A) and acetonitrile (phase B) containing 0.1% formic acid were used as the mobile phases. The elution gradient was as follows: 0 min 95% A, 5.5 min 90% A, 7.5 min 75% A, 8 min 40% A, 8.5 min 95% A, and 13 min 95% A.

### 2.6. Mineral Element Determination

The nitrogen content of the fruit pulp was determined by the perchloric acid-sulfuric acid digestion method [31]. Phosphorus content was determined by the spectrophotometric method [32]. Briefly, a 100 mg sample was oven-dried at 500 °C for 3 h and flamed to ash. The ash was then dissolved in 10% (*v*/*v*) HNO_3_ and 100 mL of 30% (*v*/*v*) HCl. Then, 10 μL of dissolved sample was mixed with deionized water, 290 mL of 0.5 M HCl, and 700 mL of Pi reaction buffer, and Pi was measured at 820 nm with a UV–Vis Spectrophotometer (Drawell DU-8600R). The potassium content was determined with a GC flame photometric detector (Agilent) [33]. The content of Ca, Mg, Fe, Zn, Mn, Ni, and Cu was determined by inductively coupled plasma atomic emission spectrometry (ICP-MS) [34]. Briefly, 1 g samples were digested by a microwave instrument (MWD-500, Metash Instrument Comp., Shanghai, China) in a mixed acid containing HClO_4_/HNO_3_ (*v*/*v* = 1/4), and then diluted to 50 mL sequentially. Metal contents were measured by using the Agilent 7900 ICP-MS.

### 2.7. Cellulose, Hemicellulose, and Pectin Determination

The content of cellulose and hemicellulose in pulp was determined by the Van Soest washing method [35]. Pectin in pulp was extracted by mixing 1.0 g of pulp with 25 mL of 95% ethanol and heating in boiling water for 30 min. After cooling, the mixture was centrifuged at 10,000× *g* for 15 min, and the supernatant was discarded. The extraction was repeated three times with 25 mL of 95% ethanol. To the original test tube containing the precipitate, 20 mL of distilled water was added, and the tube was kept in a 50 °C water bath for 30 min. The solution was filtered and diluted with distilled water to 100 mL, which was soluble pectin. To the remaining residue, 25 mL of 0.5 mol/L of sulfuric acid was added. The mixture was boiled in a water bath for 1 h, filtered, and diluted with distilled water to 100 mL to obtain the protopectin. The contents of protopectin and soluble pectin in the pulp were determined by the carbazole ethanol method [36].

One milliliter of the above extract was transferred to a 25 mL brown tube with a plug. Six milliliters of concentrated sulfuric acid were added along the wall of the tube. Next, 0.2 mL of 1.5 mol/L of carbazole ethanol solution was added. The mixture was shaken well and heated in boiling water for 20 min. The absorbance value was measured at 530 nm, and total pectin was calculated according to the formula: Total pectin = Soluble pectin (%) + Protopectin (%).

### 2.8. Polygalacturonase (PG), Pectinesterase (PE), and Cellulase Enzyme Activity Determination

The enzymes in the pulp were extracted via the sodium acetate method to determine the activity of polygalacturonase (PG), pectin esterase (PE), and cellulase [37]. PG activity is expressed in μg per gram of fresh weight (FW) fruit tissue samples per hour at 37 °C to catalyze the hydrolysis of polygalacturonic acid to produce galacturonic acid [38]. PE activity was measured using the method proposed by Chen et al. [39]. The amount of enzyme that consumed 1 μmol of NaOH per hour was defined as one unit of PE activity. Cellulase activity was defined as the amount of enzyme required to produce 1 μmol of glucose in 1 h [40].

### 2.9. Determination of Anthocyanins, Flavonoids, Limonin, and Total Phenols

One gram of peel or pulp was cut into 2–3 cm sections. Anthocyanins were extracted with 0.1 mol/L of HCl for 4 h in the dark. The optical density (OD) value of the filtrate was measured with a visible light spectrophotometer [41]. Total flavonoids in the peel or pulp were determined by the rutin colorimetric method [42]. Total phenolic compound content in the peel or pulp was determined via the Folin–Ciocalteu colorimetric method [43]. Total phenolic content was expressed in gallic acid equivalents.

The kinds and contents of flavonoids in the peel and pulp were determined by HPLC [41]. Flavonoid derivatives in the peel and pulp were extracted by grinding them into powder with liquid nitrogen and adding 5 mL of extraction solution (CH_3_OH:H_2_O:HCOOH:C_2_HF_3_O_2_ = 70:27:2:1). The extraction was performed at low temperature (4 °C) in the dark for 24 h, and the extract was filtered through a filter membrane (0.22 μm) and analyzed by HPLC.

The content of flavonoid derivatives was determined using an HPLC system (1260, Agilent) equipped with a Phenomenex Luna C18 column (4.6 mm × 150 mm, 5 μm). HPLC analysis was performed using 10 μL of the diluted supernatant [44]. The mobile phases consisted of 1% formic acid-water (A) and acetonitrile (B). The flow rate was 1.0 mL/min, and the column temperature was 30 °C. The linear gradient elution conditions were as follows: 0–42 min, 4–60% (B); 42–43 min, 60–4% (B). The content of flavonoid derivatives was determined at 280 nm by a diode array detector.

### 2.10. Dihydroflavonol-4-Reductase (DFR), Anthocyanidin Synthase (ANS), Chalcone Synthase (CHS), and Chalcone Isomerase (CHI) Activity Assay

DFR activity was measured according to a method reported by Zhou et al. [45]. One unit of DFR activity was defined as the change in the absorbance of the mixed solution at 550 nm per unit time, measured with a UV–Vis Spectrophotometer (Drawell DU-8600R), multiplied by 0.01, and the dihydroflavonol-4-reductase activity is expressed as U·g^−1^·FW (fresh weight). ANS activity was determined via the method published by Miao et al. [46]. ANS enzyme activity was calculated by measuring the absorbance value at 340 nm, and the anthocyanin synthase activity is expressed as U·g^−1^·FW.

CHS and CHI enzyme activities were determined via an ELISA kit (Shanghai Enzyme-linked Biotechnology Co., Ltd., Shanghai, China). The absorbance was measured at 450 nm with a UV-Vis Spectrophotometer (Drawell DU-8600R). One unit was defined as the amount of enzyme that catalyzed 1 mM substrate per hour.

### 2.11. Statistical Analysis

For all experiments, three independent replicates were performed. The data were statistically analyzed using two-way ANOVA with SPSS 22.0 software (IBM Comp., Chicago, IL, USA). Duncan’s multiple range test was performed to compare the means. The data were considered to be statistically significant at *p* < 0.05.

## 3. Results

### 3.1. Determination of Optimal SNP Concentration

**Figure 1:**
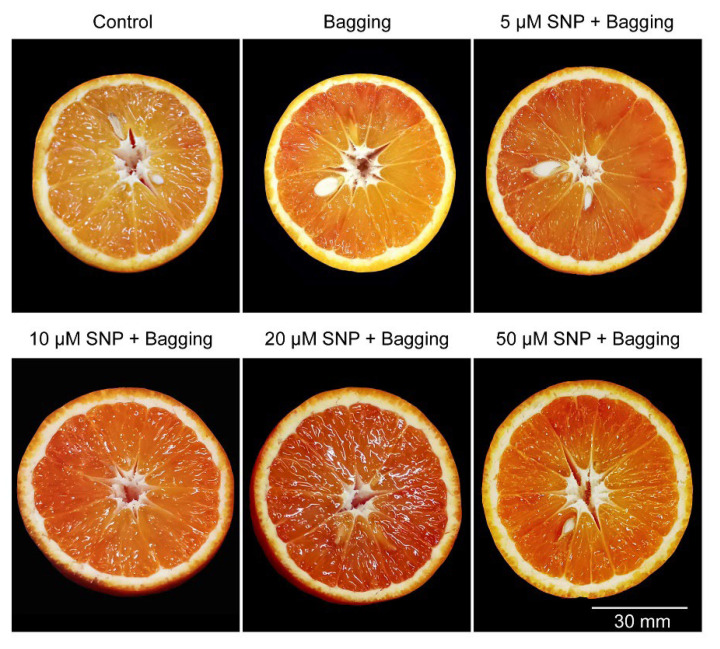
Effects of bagging treatments with 0−50 μM SNP on pulp color.

For the bagged fruit and NO-treated fruit, there were significantly more red color in the blood orange pulp than in that of the control. However, the concentrations of SNP lower than 10 μM or higher than 50 μM showed less effects to the pulp color, compared with 20 μM (Figure 1). Our results suggest that 20 μM was the optimal concentration of SNP to promote color formation in blood orange fruit.

### 3.2. Effects of Different Treatment Methods on the Physical Properties of Blood Orange

**Figure 2:**
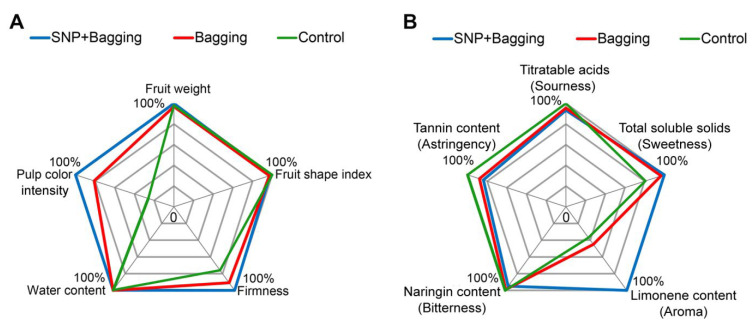
Effects of different treatments on the physical properties (single fruit weight, fruit shape index (size), firmness, water content, and pulp color) (**A**), aroma, and flavor compounds (titratable acids (sourness), total soluble solids (sweetness), limonene content (aroma), naringin content (bitterness), and tannin content (astringency)) (**B**). Relative value = measured value/max value × 100%. All measured values are shown in Table S1.

The parameters of fruit physical properties include single fruit weight, color, fruit shape index, firmness, and water content. The pulp color intensity significantly improved with NO treatment, and it was 3.8 times that of the control treatment. In addition, fruit firmness was 1.32 times that of the control treatment. The color of the bagged fruit was 3.07 times that of the control treatment, and the firmness was 1.20 times that of the control treatment. Interestingly, bagging and NO treatments had similar effects on water content, fruit weight, and fruit shape index. Compared with the bagged fruit, the NO treatment group showed improved color intensity and increased firmness of the fruit. Therefore, we inferred that it improved the physical traits of the fruit (Figure 2A and Table S1).

Sourness (characterized by titratable acids (TAs)), sweetness (characterized by TSSs), astringency (characterized by tannin content), and aroma (characterized by limonene content) were investigated. Limonin and naringin are the main sources of bitterness. The content of limonin in blood orange fruit was approximately 0.001–0.01 mg/kg, while that of naringin was approximately 240–260 mg/kg. Compared with naringin, the content of limonin was extremely low. Thus, naringin was used to characterize the fruit bitterness. NO treatment increased the TSS content in fruit and was 1.24 times that of the control treatment. It also significantly increased the content of limonene to 2.7 times that of the control treatment. Moreover, NO treatment reduced the tannin content by 16.06%, compared with the control. However, the bitter and sour taste of fruits did not change significantly in the bagged or NO-treated fruits. Therefore, NO treatment mainly improved the aroma of the fruit, followed by the sweetness of the fruit via a reduction in the astringency. Therefore, NO treatment improved physical properties of the fruit (Figure 2B and Table S1).

### 3.3. Effects of Different Treatments on Nutritional Qualities of Blood Orange Fruit

**Figure 3:**
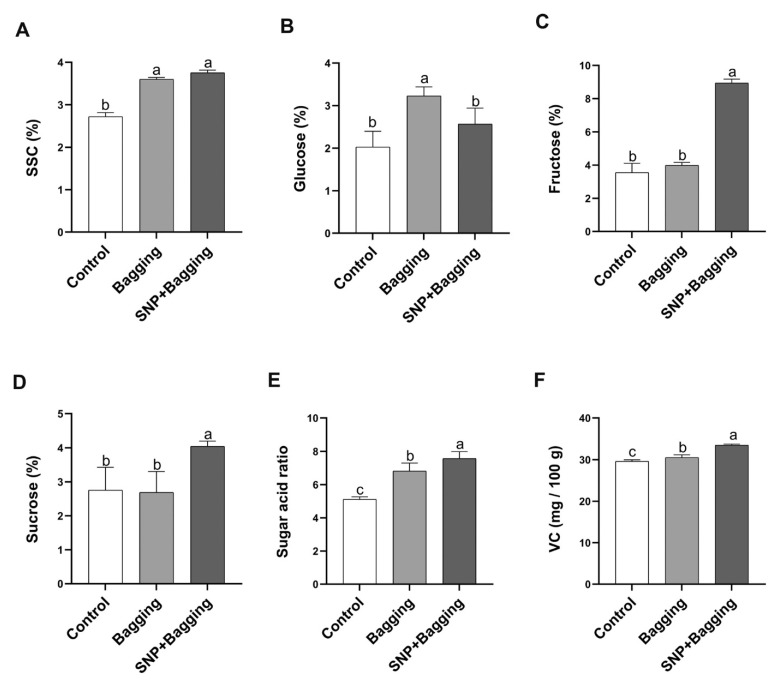
Effects of different treatments on the soluble sugar content (SSC; **A**), glucose content (**B**), fructose content (**C**), sucrose content (**D**), sugar–acid ratio (**E**), and vitamin C (VC) content (**F**) of blood orange fruit pulp. Error bars show standard deviations (*n* = 3). Different lowercase letters indicate significant differences at 0.05 (*p* < 0.05) levels.

As shown in Figure 3, bagging and NO treatment increased the content of soluble sugar (SSC) in fruits (Figure 3A). The glucose content of the bagged fruit increased by 1.59 times, compared to the control. However, NO treatment inhibited the accumulation of glucose, which was the same result as the control treatment (Figure 3B). NO treatment increased the fructose content, which was 2.52 times that of the control (Figure 3C). Moreover, NO treatment increased the sucrose content in fruit (Figure 3D). However, the contents of fructose and sucrose were similar between the bagged fruit and the control. NO treatment increased the sugar–acid ratio and VC content of fruits (Figure 3E,F), and the sugar–acid ratio of fruits treated with NO was 1.48 times higher than that of the control.

**Figure 4:**
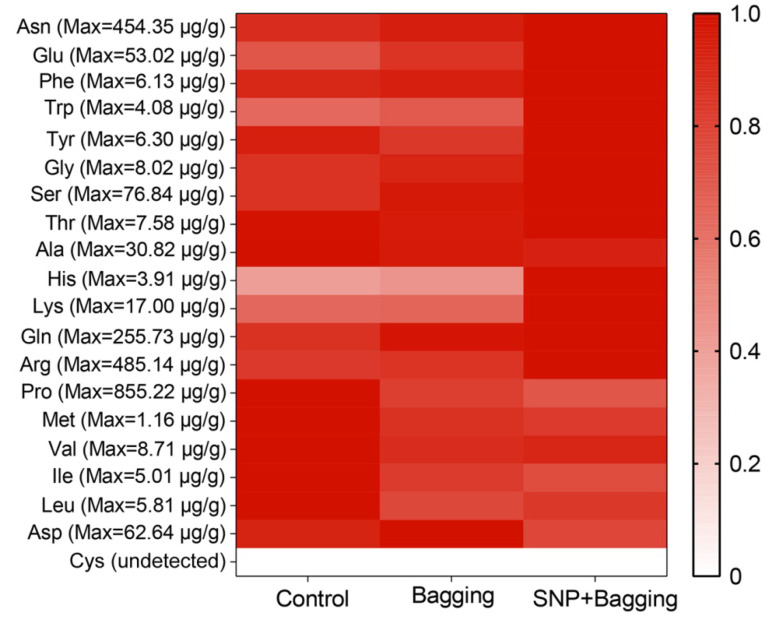
Heatmaps of amino acids in blood orange fruits after different treatments. The bar represents the relative value (measured value/max value × 100%).

NO treatment enhanced the contents of threonine, phenylalanine, glutamine, arginine, tyrosine, serine, glycine, asparagine, lysine, tryptophan, tryptophan, glutamic acid, and histidine (Figure 4). Among them, the histidine content in the fruit increased most significantly (by 1.09 and 3.23 times, respectively) after bagging and NO treatment. In the bagged and NO-treated fruit, the contents of leucine, isoleucine, aspartic acid, methionine, valine, proline, and alanine were reduced (Figure 4). In particular, the content of proline in the bagged and NO-treated fruit was dramatically reduced to 158.65 µg/g and 243.77 µg/g, respectively, less than that of the control. Moreover, cystine was not detected in the fruit.

**Figure 5:**
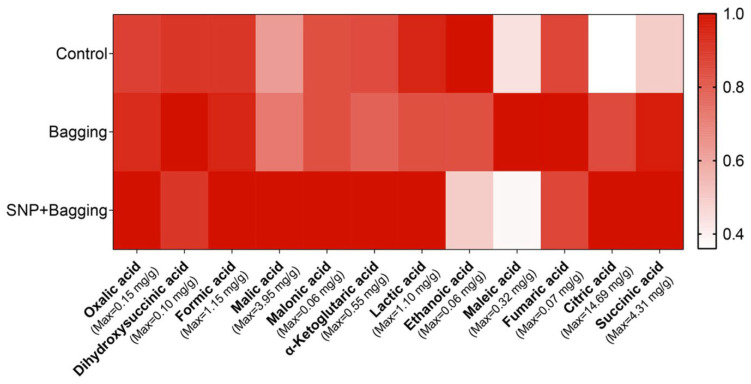
Heatmaps of organic acids in blood orange fruits after different treatments. The bar represents the relative value (measured value/max value × 100%).

The contents of oxalic acid, formic acid, malonic acid, malonic acid, a ketovalerate, lactic acid, citric acid, and succinic acid increased in the bagged and NO-treated fruit (Figure 5). Notably, the content of citric acid was 2.34 and 2.76 times that of the control, and the content of succinic acid was 1.93 and 1.99 times that of the control, respectively. In addition, the contents of malic acid, oxalic acid, formic acid, and ketovalerate also increased in the fruit. However, the content of malonic acid and lactic acid increased in NO-treated fruit but decreased in bagged fruit, similar to that of the control. Additionally, NO treatment reduced the content of acetic acid in fruit (Figure 5).

**Figure 6:**
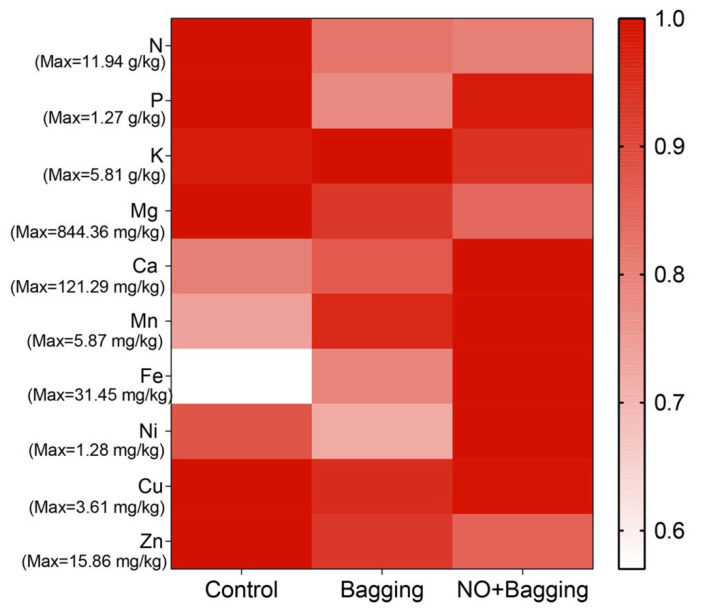
Heatmaps of mineral elements in blood orange fruits after different treatments. The bar represents the relative value (measured value/max value × 100%).

The content of Ca, Fe, Zn, and Ni increased in the bagged and NO-treated fruit (Figure 6), and the Fe content increased the most, by 1.27 and 1.75 times, respectively. The Ca content increased by 1.10 and 1.26 times in the bagged and NO-treated fruit, respectively, compared with the control. In addition, the content of N and Mg decreased, and the content of N decreased substantially. The contents of P and Mn also decreased in bagged fruit. Interestingly, there was no significant difference in the Cu content between different treatments (Figure 6).

### 3.4. Effects of Different Treatments on the Texture Qualities of Blood Orange Fruit

**Figure 7:**
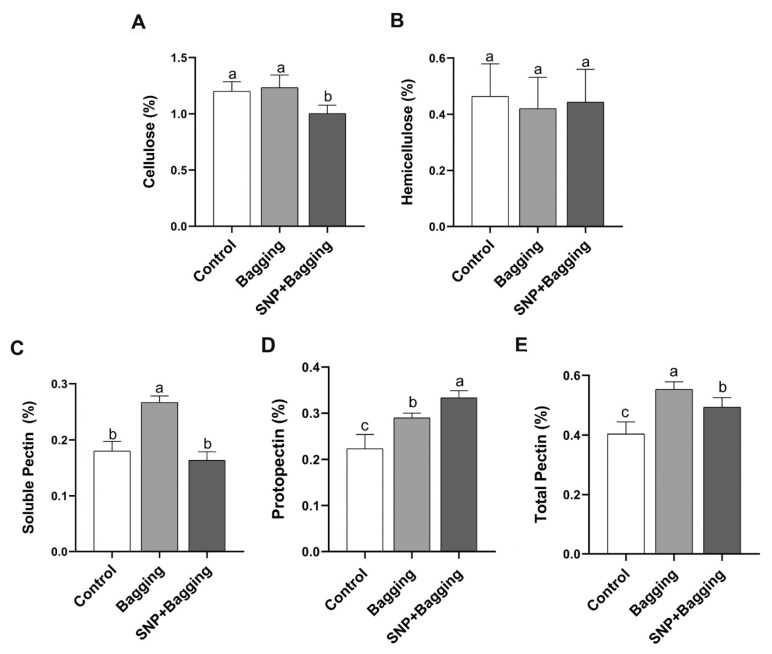
Effects of different treatments on the contents of hemicellulose (**A**), cellulose (**B**), soluble pectin (**C**), raw pectin (**D**), and total pectin (**E**) in blood orange fruit pulp. Error bars show standard deviations (*n* = 3). Different lowercase letters indicate significant differences at 0.05 (*p* < 0.05) levels.

NO treatment reduced the cellulose content in fruit, but the cellulose content of bagged fruit was consistent with that of the control (Figure 7A). The hemicellulose content was similar across the different treatments (Figure 7B). The content of soluble pectin increased by 1.46 times in bagged fruit, compared with the control, but the soluble pectin in the NO-treated fruit was similar to that of the control (Figure 7C). The original pectin content in the bagged and NO-treated fruit increased by 1.30 times and 1.48 times, respectively (Figure 7D). Moreover, the total pectin content in the bagged and NO-treated fruit also increased (Figure 7E).

NO treatment decreased the enzymatic activities of polygalacturonase (PG) and pectinase (PE) in blood orange pulp (Figure S2A,B). The cellulase activity increased by 1.27 and 1.40 times in bagged and NO-treated fruit, respectively, compared to the control (Figure S2C). This was beneficial for cellulose degradation.

### 3.5. Effects of Different Treatments on Anthocyanins, Flavonoids, and Phenols in Fruits

**Figure 8:**
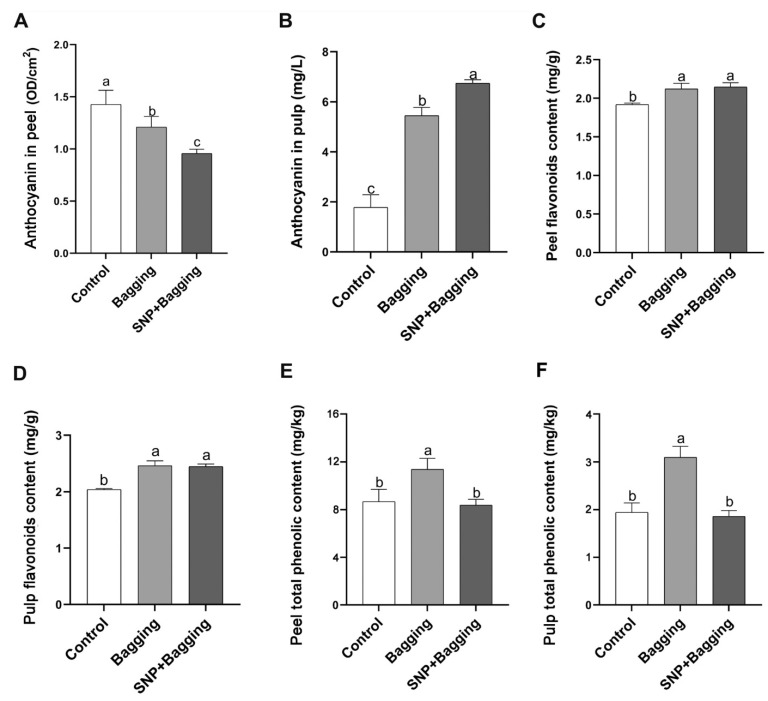
Effects of different treatments on peel anthocyanin content (**A**), pulp anthocyanin content (**B**), peel flavonoids content (**C**), pulp flavonoids content (**D**), peel total phenolic content (**E**), and pulp total phenolic content (**F**) of blood orange. Error bars show standard deviations (*n* = 3). Different lowercase letters indicate significant differences at 0.05 (*p* < 0.05) levels.

The accumulation of anthocyanins in the peel of bagged and NO-treated fruit was inhibited (Figure 8A). However, the anthocyanin content in the pulp followed the opposite trend of that in the peel. The content of anthocyanins in the pulp increased by 3.07 times and 3.80 times in the bagged and NO-treated fruit, compared with the control, respectively (Figure 8B). Bagging and NO treatment increased the total flavonoid content in the fruit peel and pulp (Figure 8C,D). The total phenol content in the pulp of bagged fruit increased by 1.31 times, compared with the control (Figure 8E). The total phenolic content in the peel or pulp of NO-treated fruit was similar to that of the control (Figure 8E).

**Figure 9:**
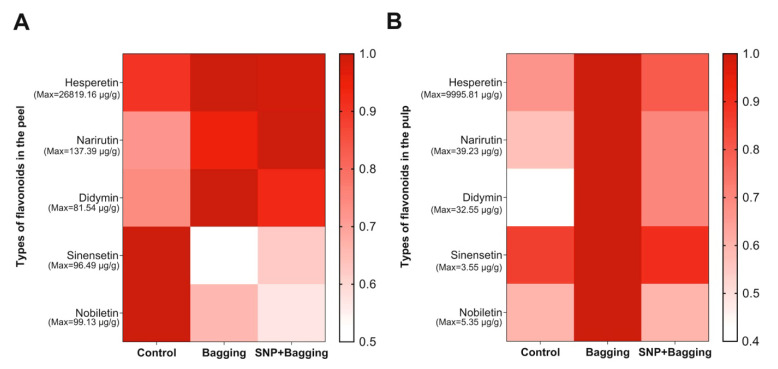
Heatmaps of flavonoids in peel (**A**) and flavonoids in pulp (**B**) after different treatments. The bar represents the relative value (measured value/max value × 100%).

The contents of hesperetin, narirutin, and didymin in the peel of bagged and NO-treated fruit increased, but the contents of sinensetin and nobiletin in the peel decreased (Figure 9A). Bagging treatment and NO treatment also increased the contents of hesperetin, narirutin, and didymin in the pulp (Figure 9B). In particular, the content of didymin was 2.53 times and 1.75 times higher than that of the control treatment in bagged and NO-treated fruit, respectively. Bagging treatment increased the contents of sinensetin and nobiletin in the pulp by 1.16 and 1.66 times, respectively. After NO treatment, the contents of sinensetin and nobiletin in pulp were similar to those in the control.

Bagging and NO treatments increased the synthesis of anthocyanins and flavonoids in the pulp (Figure S3). Chalcone synthase (CHS) activity in bagged and NO-treated fruit was 1.22 and 1.48 times that of the control, respectively (Figure S3A). Chalcone isomerase (CHI) activity was 1.14 and 1.84 times that of the control (Figure S3B), and dihydroflavonol-4-reductase (DFR) activity was 1.2 and 1.62 times that of the control (Figure S3C), respectively. The activity of anthocyanidin synthase (ANS) was 1.54 and 2.04 times that of the control (Figure S3D), respectively. Compared with the bagged fruit, NO-treated fruit exhibited improved enzymatic activity of the anthocyanin synthesis pathway.

## 4. Discussion

The L-galactose pathway is the main pathway for the synthesis of ascorbic acid (VC) in plants. L-Galactose dehydrogenase galactose1-dehydrogenase (NADP^+^) catalyzes the oxidation of L-galactose to form L-galactono-1,4-lactone (GalL), while L-galacturonic acid-1,4-lactone dehydrogenase (GalLDH) catalyzes GalL, resulting in the synthesis of VC [47]. It has been reported that the content of VC in pepper fruit treated with exogenous NO was closely related to the activity of l-galactose-1,4-lactone dehydrogenase, in which the increased GalLDH activity promoted the accumulation of VC in the fruit [48].

In this study, NO treatment significantly promoted the contents of soluble sugar, fructose, sucrose, and the sugar–acid ratio. This is consistent with the results of Shi et al. [49]. Glucose is the final product of starch hydrolysis, but starch in fruit is hydrolyzed to glucose with increasing maturity. In this study, the glucose content in pulp decreased after NO treatment, compared with the control. This is consistent with the reduction in starch granules in shoot and root starch in Arabidopsis seedlings after NO treatment [12].

Polysaccharides are the main components of plant cell walls, including cellulose, hemicellulose, and pectin [50]. Fruit softening is regulated by the degradation of the cell wall, and cell wall-modifying enzymes are responsible for regulating the fruit ripening and softening process, including pectin esterase (PE), polygalacturonase (PG), and cellulase [51]. In this study, NO treatment decreased the activities of PG and PE, but increased the activity of cellulose, which inhibited the reduction of polysaccharides in the cell wall and the degradation of protopectin. Qi et al. [52] indicated that NO inhibited the demethyl esterification of homogalacturonic acid catalyzed by PE, and delayed the further hydrolysis of the cell wall by PG. Dong et al. [53] believed that the undesirable taste and texture of citrus fruits were due to the high cellulose content in citrus pulp.

Amino acids are important indicators of fruit flavor and nutritional value, and they are closely related to human taste perception [54]. The aromatic amino acids include phenylalanine, tyrosine, and tryptophan, which are the precursors of many phenolic compounds [55]. Aspartate and glutamate contribute to the umami taste, while alanine, glycine, and serine contribute to the sweet taste [56]. In this study, the amino acid with the highest content in blood orange fruits was proline. However, NO treatment significantly reduced the content of proline and increased the contents of aromatic amino acids (phenylalanine, tyrosine, and tryptophan), umami amino acids (aspartic acid and glutamate), and sweet amino acids (glycine and serine). This may be because NO treatment increased pyruvate dehydrogenase (PDH) activity, promoted the degradation of proline to glutamate [57], and activated the shikimic acid pathway, which catalyzes the formation of shikimic acid and aromatic amino acids [55].

Organic acids are important components of fruit flavor and nutritional quality, and they have important nutritional value for the human body [58]. Citric acid, malic acid, and succinic acid were the main organic acids affecting the flavor characteristics of citrus juice [59]. In this study, the content of citric acid in blood orange fruit was highest, followed by malic acid and succinic acid. Bagging and NO treatments both improved the content of organic acids, and NO treatment had the most obvious effect. It has been reported that a high-concentration SNP treatment increased the activities of citrate synthase (CS), NAD-malate dehydrogenase (NAD-MDH), and phosphoenolpyruvate phosphatase (PEPP) and promoted the accumulation of citric acid in citrus roots with aluminum deficiency [58]. SNP treatment also increased the toxicity of succinic acid, propionic acid, butyric acid, oxalic acid, formic acid, malic acid, malonic acid, and benzoic acid under a boron stress in wheat buds and roots [60].

For all mineral elements, the Ca^2+^ content in the fruit increased most significantly after the NO treatment. Ca is a key regulator of thickness and mechanical strength of the cell wall, which determine the softness and slagging of the fruit [61]. Demethylated homogalacturonan (HGA) combines with Ca^2+^ to form pectate calcium, to improve cell wall resistance to decomposition and increase mechanical firmness [62]. Earlier studies found that low concentrations of Ca^2+^ made the cell wall softer and more prone to rupture, while high concentrations made the cell wall stiffer and less plastic [61]. NO elevated the activity of H^+^-ATP (adenosine triphosphate) in the plasma membrane and hence increased the uptake of Ca in roots and shoots under environmental stress [62,63]. Thus, the improved fruit firmness by NO treatment found in this study may be associated with the increased Ca content in blood orange pulp.

Citrus fruit pigment formation is closely related to anthocyanin accumulation. In this study, NO treatment significantly increased the content of total flavonoids and anthocyanin in the pulp and upregulated the activity of key enzymes of the anthocyanin synthesis pathway (CHS, CHI, DFR, and ANS) to promote anthocyanin accumulation [64]. It has been confirmed that anthocyanin synthetases (ANS) were important links in the formation of colored anthocyanins from colorless anthocyanins [65]. The upregulation of genes encoding CHS, CHI, DFR, and ANS has been found to promote anthocyanin accumulation in strawberry fruits [64]. Moreover, NO could increase ROS accumulation and induce anthocyanin biosynthesis subsequently [66,67].

Limonene is a ubiquitous monocyclic monoterpene essential oil in plants with a special aroma resembling that of lemon [68]. Monoterpenes form isopentenyl pyrophosphate (IPP), the central precursor of terpenoids, and dimethylallyl pyrophosphate (DMAPP) after catalysis by the MEP (2-C-methyl-D-erythritol4-phosphate) and DXP (1-deoxy-D-xylulose 5-phosphate) pathways from acetyl coenzyme A, pyruvic acid, and glyceraldehyde-3-phosphate [68]. IPP and DMAPP are condensed by geranyl diphosphate synthase (GPPS) to produce geranyl diphosphate (GPP) and neryl diphosphate (NPP), the precursors of monoterpenes, and NPP is catalyzed by citrate synthase to synthesize limonene monoterpenes. NPP is catalyzed by lemon synthase to synthesize monocyclic monoterpene limonene [68]. In this study, NO treatment increased the content of limonene in the peel and increased the aroma of the peel. Consistent with a previous report [12], NO inhibited the conversion of pyruvate to acetyl coenzyme A, which is a key step in the terpenoid biosynthesis pathway.

Tannin is the main cause of astringency in fruit [69]. Our results show NO treatment reduced the content of tannin in the pulp and reduced astringency. Studies have found that polyphenol oxidase (PPO) and peroxidase (POD) could catalyze phenolic compounds to form quinones and concentrate tannins into brown-colored polymers. Exogenous NO treatment inhibited PPO and POD activities in fresh-cut chestnut pulp and delayed tannin accumulation [70].

## 5. Conclusions

In a nutshell, exogenous NO treatment improved the color and firmness of blood orange pulp, as well as the physical properties of the fruit. Astringency was minimized by reducing the content of tannin in the pulp. NO treatment increased the content of limonene (aroma) in the peel and soluble solid content (sweetness) in the pulp, and improved physical properties of the fruit. Therefore, NO treatment enhanced blood orange fruit qualities and economic values. Although SNP is an inexpensive chemical and has been widely used in mammals and plants, it has been reported to release toxic cyanide and reduce the photochemical activity of photosystem II during its photolysis [71,72]. New NO donors with both high releasing efficiency and high safety should be developed. NO is tightly correlated with nitrate metabolism. Appropriate NO treatments combined with nitrogen management are worth further studies.

## Data Availability

Data are contained within the article.

## Supplementary Materials

The following supporting information can be downloaded at: https://www.mdpi.com/article/10.3390/foods11152218/s1, Table S1. Measured values of Figure 2; Figure S1. Bagging and NO+Bagging treatments to blood orange fruits; Figure S2. Effects of different treatments on polygalacturonase (PG; A), pectinase (PE; B), and cellulase (C) activities in blood orange fruits; Figure S3. Effects of different treatments on the activities of chalcone synthase, CHS (A), chalcone isomerase, CHI (B), dihydroflavonol-4-reductase, DFR (C) anthocyanin synthase, and ANS (D) in anthocyanin biosynthesis pathway of blood orange pulp.
