# Supplementary material for: Beneficial Effects of Sodium Nitroprusside on the Aroma, Flavors, and Anthocyanin Accumulation in Blood Orange Fruits
Source: Foods. 2022 Jul 26;11(15):2218. doi: 10.3390/foods11152218 (PMC9329794; doi:10.3390/foods11152218)
Supplement: Supplementary file 1 [file foods-11-02218-s001.zip › foods-1821752-supplementary.pdf]

Table S1. Measured values of Figure 2.

Measured values of Radar chart A			
Parameter (unit)	Control	Bagging	SNP+Bagging
Fruit weight (g)	160.0 ± 15.3	158.1 ± 7.1	163.1 ± 4.0
Fruit shape index	0.94 ± 0.03	0.91 ± 0.06	0.92 ± 0.04
Firmness (Kg/cm^2)	1.58 ± 0.10	1.89 ± 0.12	2.08 ± 0.16
Water content (%)	84.3% ± 0.5%	84.6% ± 0.3%	84.4% ± 0.6%
Red color intensity of the pulp (% of Max)	26.2 ± 2.1%	80.7 ± 3.5 %	100%

Measured values of Radar chart B			
Parameter (unit)	Control	Bagging	SNP+Bagging
Titratable acids (TA; %)	0.53% ± 0.03%	0.53% ± 0.03%	0.50% ± 0.02%
Total soluble solids (TSS; %)	8.76 ± 0.10	10.47 ± 0.10	10.56 ± 0.48
Limonene (mg/g)	5.08 ± 0.78	6.13 ± 0.40	13.65 ± 1.19
Naringin (mg/kg)	254.8 ± 10.1	257.8 ± 5.8	256.0 ± 16.0
Tannin (mg/kg)	79.29 ± 3.90	69.70 ± 7.06	66.56 ± 2.16

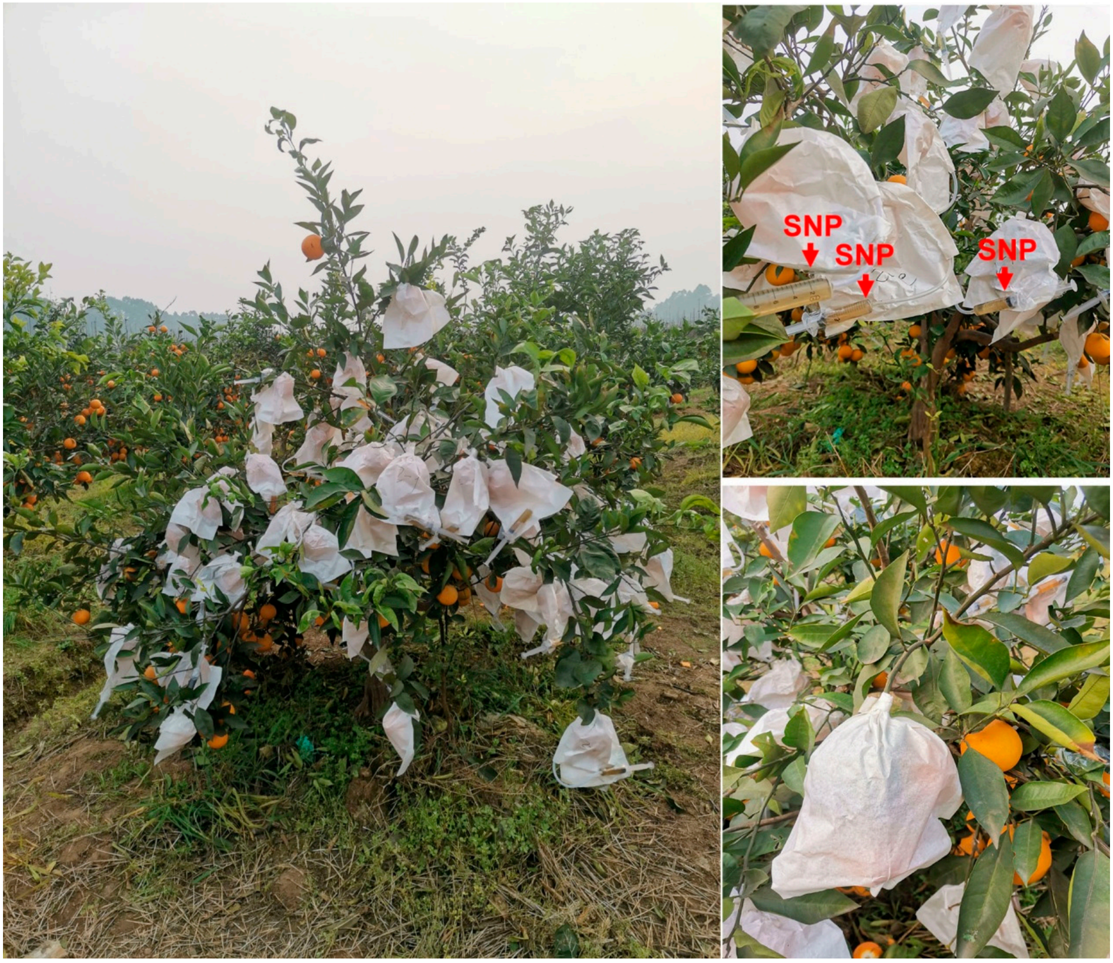

Figure S1. Bagging and NO+Bagging treatments to blood orange fruits.

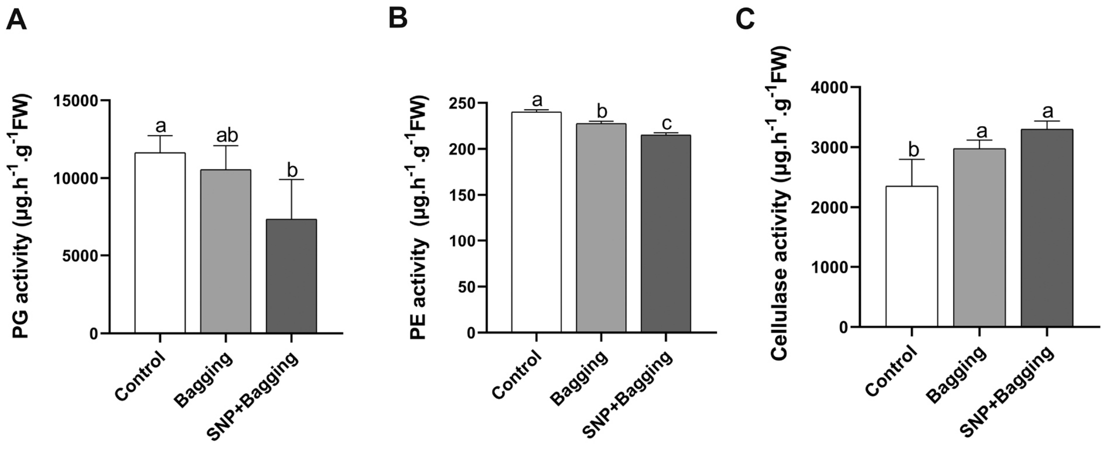

Figure S2. Effects of different treatments on polygalacturonase (PG; A), pectinase (PE; B) and cellulase (C) activities in blood orange fruits. FW, fresh weight. Error bars show standard deviations (n = 3). Different lowercase letters indicate significant differences at 0.05 ($P < 0.05$) levels.

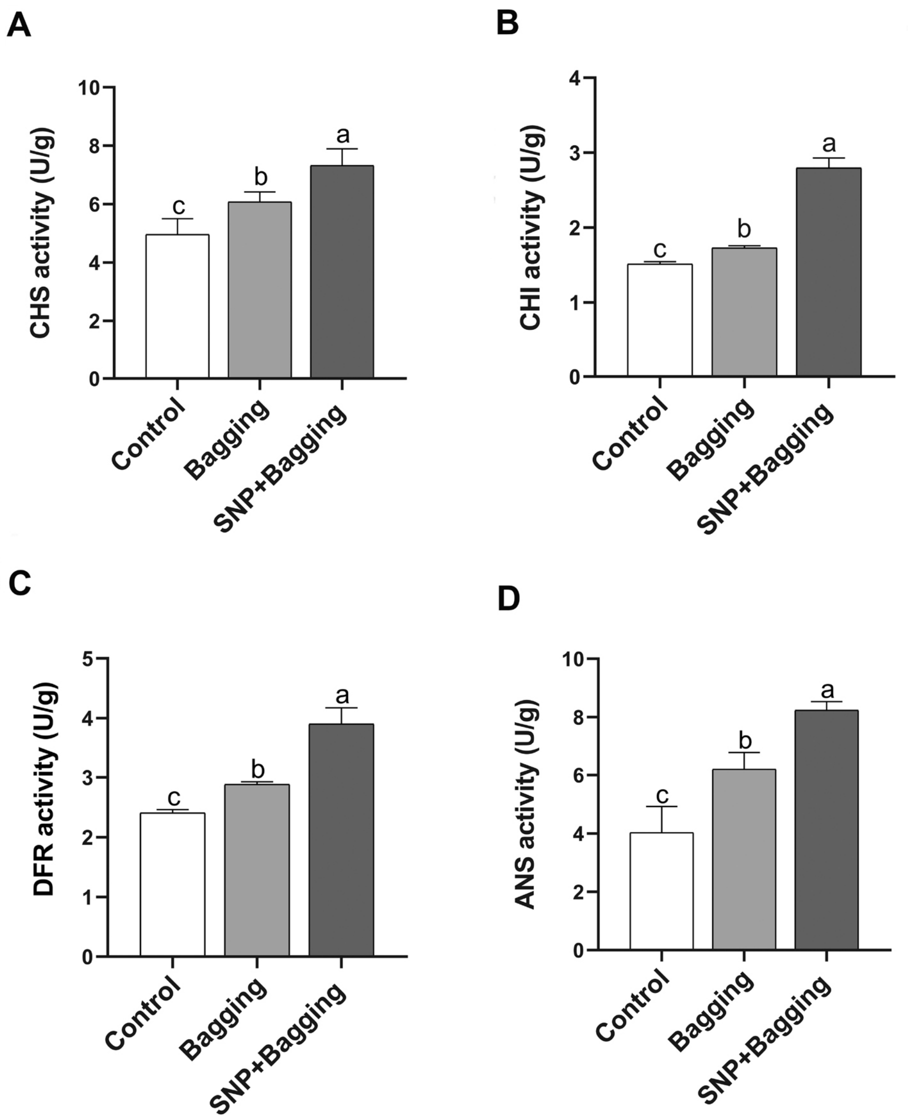

Figure S3. Effects of different treatments on the activities of chalcone synthase, CHS (A), chalcone isomerase, CHI (B), dihydroflavonol-4-reductase, DFR (C) anthocyanin synthase, ANS (D) in anthocyanin biosynthesis pathway of blood orange pulp. Error bars show standard deviations (n = 3). Different lowercase letters indicate significant differences at 0.05 ($P < 0.05$) levels.
